# Quantifying asymptomatic infection and transmission of COVID-19 in New York City using observed cases, serology, and testing capacity

**DOI:** 10.1073/pnas.2019716118

**Published:** 2021-02-10

**Authors:** Rahul Subramanian, Qixin He, Mercedes Pascual

**Affiliations:** ^a^Department of Ecology and Evolution, Biological Sciences Division, University of Chicago, Chicago, IL 60637;; ^b^Santa Fe Institute, Santa Fe, NM 87501

**Keywords:** COVID-19, testing submodel, asymptomatic transmission, epidemiological model, epidemiological parameter estimates

## Abstract

The contributions of asymptomatic infections to herd immunity and community transmission are key to the resurgence and control of COVID-19, but are difficult to estimate using current models that ignore changes in testing capacity. Using a model that incorporates daily testing information fit to the case and serology data from New York City, we show that the proportion of symptomatic cases is low, ranging from 13 to 18%, and that the reproductive number may be larger than often assumed. Asymptomatic infections contribute substantially to herd immunity, and to community transmission together with presymptomatic ones. If asymptomatic infections transmit at similar rates as symptomatic ones, the overall reproductive number across all classes is larger than often assumed, with estimates ranging from 3.2 to 4.4. If they transmit poorly, then symptomatic cases have a larger reproductive number ranging from 3.9 to 8.1. Even in this regime, presymptomatic and asymptomatic cases together comprise at least 50% of the force of infection at the outbreak peak. We find no regimes in which all infection subpopulations have reproductive numbers lower than three. These findings elucidate the uncertainty that current case and serology data cannot resolve, despite consideration of different model structures. They also emphasize how temporal data on testing can reduce and better define this uncertainty, as we move forward through longer surveillance and second epidemic waves. Complementary information is required to determine the transmissibility of asymptomatic cases, which we discuss. Regardless, current assumptions about the basic reproductive number of severe acute respiratory syndrome coronavirus 2 (SARS-Cov-2) should be reconsidered.

## Significance

As health officials face another wave of COVID-19, they require estimates of the proportion of infected cases that develop symptoms, and the extent to which symptomatic and asymptomatic cases contribute to community transmission. Recent asymptomatic testing guidelines are ambiguous. Using an epidemiological model that includes testing capacity, we show that many infections are nonsymptomatic but contribute substantially to community transmission in the aggregate. Their individual transmissibility remains uncertain. If they transmit as well as symptomatic infections, the epidemic may spread at faster rates than current models often assume. If they do not, then each symptomatic case generates, on average, a higher number of secondary infections than typically assumed. Regardless, controlling transmission requires community-wide interventions informed by extensive, well-documented asymptomatic testing.

Since the emergence of the novel coronavirus in December 2019 (1), the COVID-19 pandemic has resulted in over 16 million cases and 600,000 deaths worldwide (2). Schools and universities in the United States are gradually reopening amid concerns that a second wave of the epidemic may reemerge in the fall and winter of 2020.

As they craft testing policies and intervention strategies to mitigate a second wave, public health officials need to better understand the role that symptomatic and asymptomatic individuals play in the community transmission of COVID-19 and in the development of herd immunity to the disease. However, fundamental epidemiological questions remain poorly understood, including what fraction of cases are symptomatic and how well asymptomatic cases can transmit relative to symptomatic ones. These questions are especially urgent given ambiguity in recent Centers for Disease Control and Prevention (CDC) guidelines regarding the testing of asymptomatic individuals (3).

Answering these questions can also provide further insight on the basic reproductive number of severe acute respiratory syndrome coronavirus 2 (SARS-CoV-2), and how the virus would spread in a population in the absence of interventions. This number, known as $R_{0}$, is defined as the mean number of secondary cases arising from a primary case in the absence of immunity, and is estimated on the basis of a particular epidemiological model. Mathematical models for the population dynamics of COVID-19 incorporate different features such as asymptomatic and presymptomatic transmission, superspreading, or heterogeneity in susceptibility. A considerable range of $R_{0}$ estimates has been reported, ranging from at least 1.5 (4) to 5.7 (5) in Wuhan. A much narrower range, between two and three, is frequently cited in the popular press, or assumed when simulating models (6) or fitting these to data (7, 8). This assumption may be based on the dynamics of COVID-19 in regions that implemented interventions early (9–13). A more precise estimate of $R_{0}$ from a city where substantial transmission was occurring prior to intervention, such as New York City, would provide a relevant baseline. Furthermore, if “superspreading” by a small fraction of symptomatic infections fuels COVID-19 transmission, a precise estimate of the mean number of secondary cases arising from such an individual may be just as valuable. A model that precisely estimates the fraction of symptomatic cases may help epidemiologists discern whether either the overall or symptomatic reproductive numbers are higher than assumed.

The probability that a COVID-19 infection is symptomatic is difficult to estimate (14), and a wide range of values have been suggested (14–16). Estimates from cruise ship outbreaks (17), Wuhan evacuees (18), long-term care facilities (19), and contact tracing of index cases (15) may not be representative of the general population. Increases in the testing capacity for COVID-19 over time (9, 20, 21) make population-level estimation of this probability difficult due to confounding with other parameters such as the reporting, hospitalization, and fatality rates. When the testing capacity is limited in the early stages of an outbreak, severe cases are more likely to be tested, which can bias estimates of the probability that an infection is symptomatic and of the fatality rate. Changes in testing capacity over time also confound the definition itself of asymptomatic individuals in transmission models, when these are not differentiated from unreported cases. These changes can also bias the reported deaths attributed to COVID-19.

These challenges can be improved upon by explicitly incorporating changes in testing capacity into an epidemiological process model. While some early models of the COVID-19 outbreak in Wuhan attempted to take into account changes in testing capacity (21) or differences in reporting rate during periods of the epidemic (9), the limited information on these trends in Wuhan meant that they had to be estimated on a coarse temporal scale (2- to 3-wk intervals) and had to be inferred along with other parameters in the model. In the United States, many states and municipalities such as New York City (22, 23) have published daily estimates of the number of total COVID-19 tests conducted, together with the number of positive COVID-19 tests. While these data are often used by public health officials to gauge the spread of the COVID-19 outbreak, they have yet to be incorporated explicitly into epidemiological models.

We present an epidemiological model that incorporates RT-PCR testing as an integral process informed by empirical levels. The explicit consideration of testing allows us to clearly define asymptomatic individuals as those that will never transition to displaying symptoms, and to differentiate them from those who have been unreported because they were not tested. We fit the model to PCR-confirmed COVID-19 cases in New York City, using publicly available data provided by the New York State Department of Public Health (23). The resulting model can clearly delineate symptomatic and asymptomatic infections independently from the reporting rate. We subsequently fit the model to estimates of prior exposure obtained from a recent serological study in New York City (24) to further constrain inference results.

Our model obtains a precise estimate for the symptomatic proportion of COVID-19 cases. We show that most COVID-19 infections are asymptomatic, and that these asymptomatic infections together with presymptomatic ones substantially drive community transmission, contributing 50% or more of the total force of infection. Furthermore, depending on the transmissibility of individual asymptomatic cases relative to symptomatic ones, either the overall reproductive number or the symptomatic reproductive number may be higher than typically assumed. Our results highlight the importance of testing and contact tracing of asymptomatic individuals, and of making these data publicly available as health officials prepare for and manage a second wave.

## Results

**Fig. 1.**
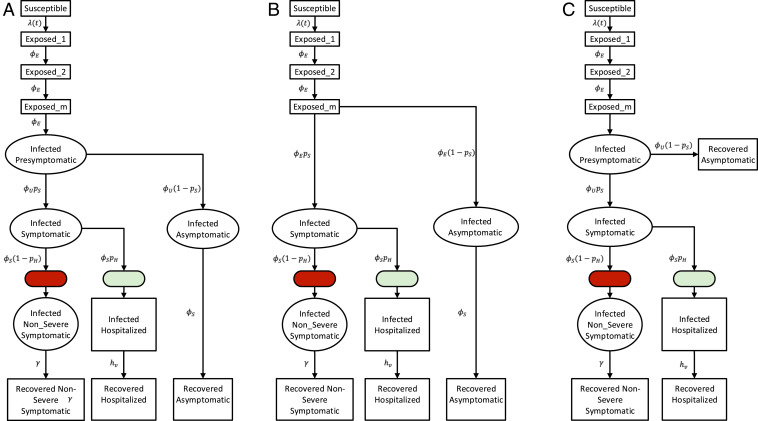
Model diagrams. (*A*) The full SEPIAR model used for inference. The model is an extension of an SEIR formulation that considers both presymptomatic transmission (from compartment P) and asymptomatic transmission (from compartment A). (*B*) When the strength of presymptomatic transmission $b_{p}$ is set to zero, the SEPIAR model reduces to the SEIAR model. Since we assume that $\phi_{U}=\phi_{E}$, when $b_{p}=0$, the infectious presymptomatic compartment behaves like an additional exposed compartment. (*C*) When the strength of asymptomatic transmission $b_{a}$ is set to zero, the SEPIAR model reduces to the SEPIR model. Individuals in the asymptomatic infectious compartment (A) make no contribution to the force of infection, so asymptomatic individuals essentially recover after leaving the presymptomatic period (P). In all three panels, circular/elliptical compartments contribute to the force of infection, while rectangular compartments do not. The green ellipse denotes the point at which severe/hospitalized COVID patients are sampled and enter the testing queue for severe cases, while the red ellipse denotes the corresponding entry point for the queue for nonsevere symptomatic cases.

We present a stochastic epidemiological model (Fig. 1) that explicitly incorporates daily changes in testing capacity and the lag between sampling and testing (see *Materials and Methods*). The underlying model, referred to hereafter as the SEPIAR model (Fig. 1*A*), has a susceptible–exposed–infectious–recovered (SEIR) structure with compartments for both severe (hospitalized) and nonsevere symptomatic infections as well as presymptomatic (P) and asymptomatic (A) infections (thus SEPIAR). We also consider two nested simplified versions: one with no presymptomatic transmission (the SEIAR model; Fig. 1*B*) and one with no asymptomatic transmission (the SEPIR model; Fig. 1*C*). By varying specific parameters weighting the transmission rate of P and A relative to that of symptomatic individuals, we can continuously move across these two extreme structures. Daily reports of the number of tests conducted in New York City are fed in as a covariate in the testing submodel (*SI Appendix*). The model takes into account CDC priorities in sampling and testing: All hospitalized cases are sampled and eventually tested, while nonsevere symptomatic individuals are sampled and tested only if excess capacity is available at the time of sampling. We also incorporate the retesting of hospitalized individuals as they leave the hospital. This model is fit to observed cases in New York City from March 1, 2020 to June 1, 2020 and to serological estimates of herd immunity in New York City from March 8, 2020 to April 19, 2020 (*Materials and Methods* and *SI Appendix*). We compare the full model with the two nested simplified versions. Although all three model structures are supported by the case data, the model with no asymptomatic transmission is not supported when these data are considered in conjunction with serology information (*SI Appendix*, Table S2).

To evaluate the strength of transmission in asymptomatic cases relative to symptomatic cases, we construct a Monte Carlo profile using the full SEPIAR model (*SI Appendix*, Fig. S5). We isolate parameter combinations from the profile that are supported by the case and serology data, and examine the values of those combinations. Particular parameters of interest that we focus on include the proportion of cases that are symptomatic, $p_{S}$, the ratio of the transmission rate of asymptomatic individuals to that of symptomatic individuals, $b_{a}$, and the reproductive numbers. We use $R_{0}$ to denote the symptomatic reproductive number (i.e., the mean number of secondary cases arising from each primary symptomatic case), and use $R_{0_{NGM}}$ to denote the overall reproductive number for the model (i.e., the mean number of cases arising from a primary infection, where the average considers all types of infections).

**Fig. 2.**
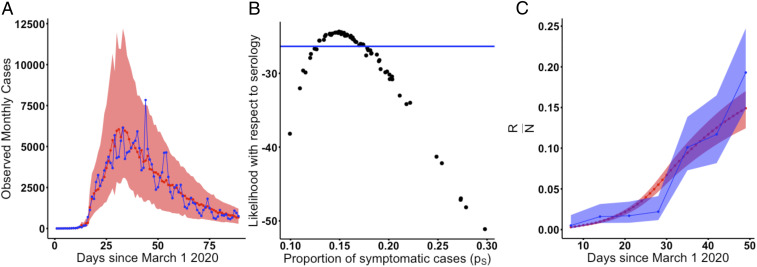
The probability of symptomatic infection. (*A*) Simulated vs. observed cases from the profile of the asymptomatic transmission strength ($b_{a}$) using the SEPIAR model. The red line is the median from 100 simulations using the MLE, while the red shaded region denotes the 2.5 to 97.5% quantiles across 100 simulations from all parameter combinations within two log-likelihood units of the profile MLE. Likelihoods here are with respect to case data. The observed daily case counts are denoted by the blue line. (*B*) Model likelihood as a function of the proportion of cases that are symptomatic ($p_{S}$) for each parameter combination from *A*. The *y* axis shows the likelihood for that parameter combination with respect to serology data. All parameter combinations above the blue line have likelihoods within two log-likelihood units of the MLE (defined with respect to serology). This corresponds to a range of values for $p_{S}$ of approximately 13 to 18%. (*C*) Comparison of observed vs. simulated estimates of herd immunity in the population from parameter combinations supported by both case and antibody data (all points above the blue line in *B*). The red line denotes the median value of herd immunity (the proportion of the population that has recovered [*R*/*N*]) at that point in time in 100 simulations from the MLE parameter combination. The red shaded region denotes the 2.5 to 97.5% quantiles for these simulations from all parameter combinations within two log-likelihood units of the MLE with respect to serology (all parameter combinations above the blue line in *B*). The blue line denotes estimates of herd immunity from a recent serological survey in New York City (24). The blue shading denotes 95% CIs for those serology estimates using the methods of ref. 24.

The proportion of COVID-19 cases that are symptomatic is well identified, with a CI ranging from 12.9 to 17.4% (Fig. 2). Although a wide range of parameter combinations for the proportion of symptomatic infection are supported by the case data on its own, a much narrower estimate is obtained when the case and serology data are considered together (Fig. 2 *A* and *B*). Within this range, estimates of herd immunity are consistent with the dynamics of observed serology (Fig. 2*C*), in particular, the rapid rise in seroprevalence over March and April 2020. We validated the inference pipeline by fitting the model to two simulated trajectories from two parameter combinations that are both supported by the case, serology, and testing data but correspond to regimes with strong or weak asymptomatic transmission. As shown in *SI Appendix*, Fig. S11*B*, models fit to both of these trajectories instead of observed cases are able to accurately estimate and recover the proportion of symptomatic cases used in the simulations.

**Fig. 3.**
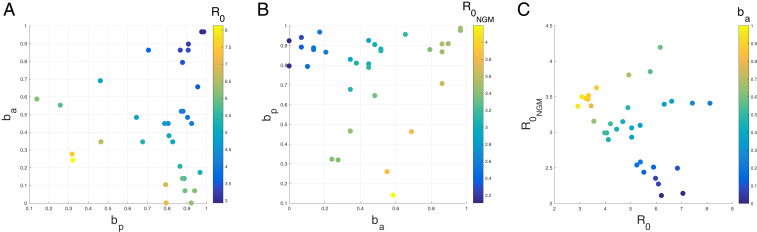
Plots of (*A*) the reproductive number of symptomatic individuals ($R_{0}$) and (*B*) the overall reproductive number ($R_{0_{NGM}}$), as a function of the relative strength of presymptomatic transmission ($b_{p}$) and the relative strength of asymptomatic transmission ($b_{a}$). Each point represents one parameter combination within two log-likelihood units of the MLE (with respect to serology) from the $b_{a}$ profile. (*C*) Plot of the overall reproductive number vs. the reproductive number in symptomatic individuals for the same points colored by $b_{a}$. The black arrows show the direction of increasing strength of asymptomatic transmission ($b_{a}$) and presymptomatic transmission ($b_{p}$). For this same plot, except colored by the strength of presymptomatic transmission ($b_{p}$), see *SI Appendix*, Fig. S6. For ease of plotting, we exclude two parameter combinations which had very low relative rates of presymptomatic transmission (i.e., $b_{p}$ was lower than 0.020). The two outlier combinations had high reproductive numbers ($R_{0}=17.77,R_{0_{NGM}}=3.95$ and $R_{0}=4.97,R_{0_{NGM}}=4.37$). These outliers are included in *SI Appendix*, Fig. S7.

The overall reproductive number or symptomatic reproductive number may be larger than is often assumed. From our profile of the relative asymptomatic transmission rate $b_{a}$, we identify two main regimes of transmission that are supported by both the case and serology data (Fig. 3), in which either $R_{0}\;orR_{0_{NGM}}$ is higher than the two to three range often assumed for COVID-19. Notably, we find no parameter combinations in which both reproductive numbers are below three and fall within this range.

**Fig. 4.**
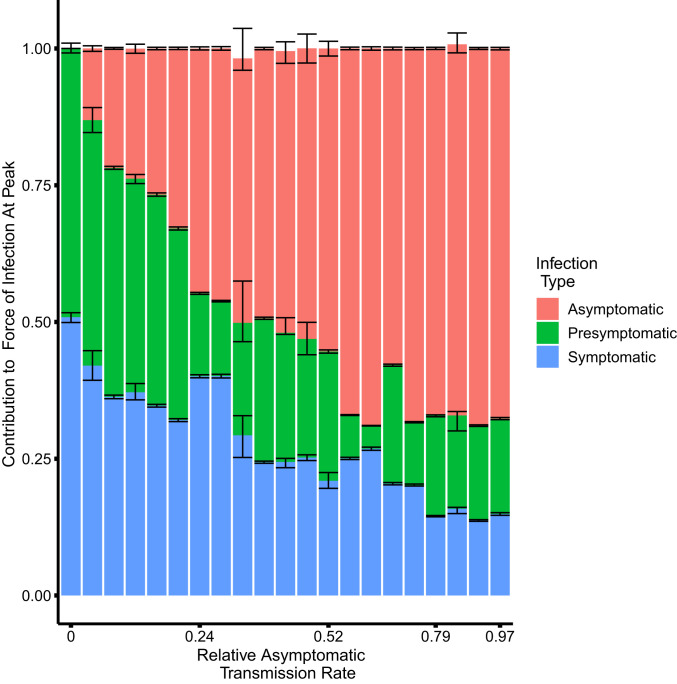
The contribution to the force of infection at the peak of the outbreak on April 14, 2020 from symptomatic, asymptomatic, and presymptomatic infections under different relative asymptomatic transmission rates $b_{a}$. For each parameter combination from the fitted SEPIAR model supported by case and serology data (corresponding to the points in Fig. 3), we simulate 100 trajectories and calculate the proportion of the overall force of infection on April 14, 2020 that is due to asymptomatic, symptomatic, and presymptomatic infections. We pool trajectories from all parameter combinations that have the same value of $b_{a}$, and calculate the median, 2.5%, and 97.5% quantiles for each infection class and value of $b_{a}$. The colored bars represent, for each infection class, the median proportion of its contribution to the force of infection (and hence may not sum exactly to one). The error bars represent the corresponding 2.5% and 97.5% quantiles. Versions of this plot calculated 4 wk before and 4 wk after the peak can be found in *SI Appendix*, Fig. S9. We excluded two outlier parameter combinations that had extremely low relative rates of presymptomatic transmission (i.e., where $b_{p}$ was less than 0.02).

In the first regime, asymptomatic individuals transmit at almost the same rate as symptomatic individuals. That is, $b_{a}$ is large, even close to one in some parameter combinations. The overall reproductive number takes on values between 3.2 and 4.4, and asymptomatic cases substantially contribute to the overall force of infection (Fig. 4).

In the second regime, asymptomatic individuals transmit at very low rates relative to symptomatic individuals, with estimates of $b_{a}$ close to zero or, in some parameter combinations, even equal to zero. Concomitantly, the symptomatic reproductive number is much higher than frequently assumed, taking on values between 3.9 and 8.1. Nevertheless, even in this regime, presymptomatic and asymptomatic infections together contribute at least 50% of the overall force of infection at the peak of the outbreak.

In both regimes, presymptomatic individuals transmit at almost the same rate as symptomatic individuals, with estimates of $b_{p}$ close to one, also making a substantial contribution to the overall force of infection (Fig. 4).

We also observe a third regime in which both reproductive numbers are higher than assumed, but, in this regime, presymptomatic individuals transmit at a very low rate, with $b_{p}$ close to zero. Several combinations in this regime can be observed in the top right corner of Fig. 3*C* and in *SI Appendix*, Fig. S7. This is also the regime obtained in *SI Appendix*, Fig. S6 if one uses the SEIAR model, which assumes that presymptomatic individuals do not transmit (i.e., $b_{p}$ is fixed at zero). Given previous evidence of presymptomatic transmission of COVID-19 (25, 26), we focus on the two regimes which incorporate substantial presymptomatic transmission.

**Fig. 5.**
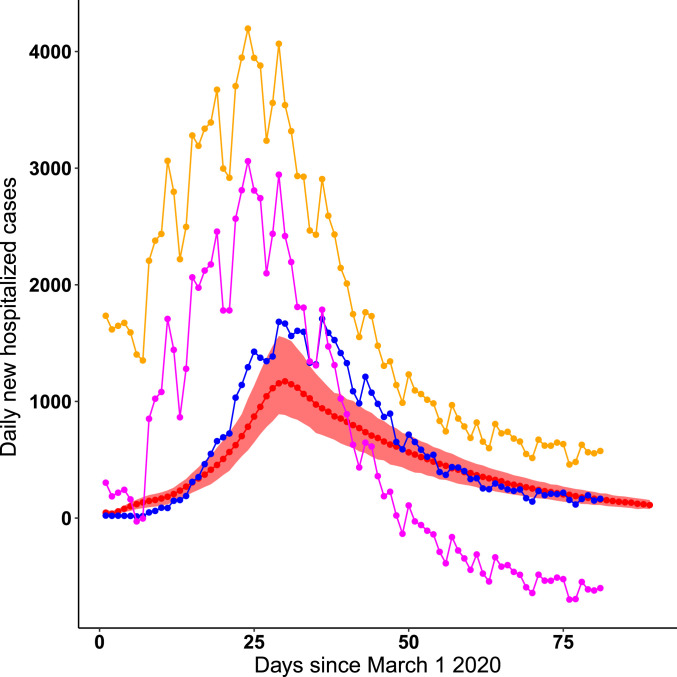
Comparison of daily COVID hospitalizations under the model with observed COVID hospitalizations in New York City and emergency department respiratory syndrome surveillance reports. The red line represents the median daily case hospitalizations from 100 simulations from the parameter combination with the highest likelihood with respect to serology from the $b_{a}$ profile. The red shading represents the bounds of the 2.5% and 97.5% quantiles across all parameter combinations from the $b_{a}$ profile that are supported by case and serology data. The blue line shows observed COVID daily hospitalizations in New York City. The yellow line denotes daily reports of respiratory illness from syndrome surveillance in New York City emergency departments, while the pink line denotes anomalous respiratory surveillance reports compared to previous years.

In line with previous studies (27), we estimate a large value for the initial number of infected and incubating individuals with COVID-19 in New York City at the start of the simulation on March 1. Parameter combinations that were supported by the case and serology data ranged from 9,000 to 18,000 initial infected individuals and 44,000 to 72,000 exposed individuals. A key question to consider when evaluating the plausibility of this magnitude of undetected infections is whether it is consistent with no signal of an anomalous number of hospitalizations. In other words, would this large rise in early infections result in a corresponding rise in COVID hospitalizations that may not have been detected as COVID related? We examine this question by comparing simulated daily hospitalizations from our fitted model with observed COVID-19 daily hospitalizations in New York City, as well as with syndrome surveillance reports of respiratory illness from emergency departments in New York City hospitals (Fig. 5), which we can use as an indicator for a rise in undetected hospitalizations. We show that a scenario with a large number of initial infections on March 1 is indeed consistent with the time at which observed COVID-19 hospitalizations peak, providing further support for this contention. We also find that the imposition of social distancing on March 17 and the stay-at-home order on March 22 in New York City resulted in a substantial decrease in the initial transmission rate. Parameter estimates for the ratio of the postintervention transmission rate to the preintervention transmission rate ($b_{q}$) ranged from 0.134 to 0.240, corresponding to a 75.98 to 86.62% reduction in the strength of transmission after the intervention.

Testing strategies and capacity can substantially influence estimates of the infection fatality ratio, or IFR (*SI Appendix*, Fig. S12). This metric of outbreak severity is generally defined as the total number of deaths divided by the total number of cases. In practice, this ratio is calculated by dividing the total number of confirmed deaths by the total number of observed cases. However, depending on the testing strategy used and the testing capacity available, not all cases will be observed. Using parameters from the fitted SEPIAR model that are supported by case and serology data, we generate a range of IFRs that would be expected under two different testing strategies. Since we do not model deaths from COVID-19 hospitalized patients in our model, we estimate the proportion of hospitalizations that result in death using confirmed COVID-19 hospitalizations and deaths in New York City during the study period. In the first testing strategy, all cases are observed; in the second one, all symptomatic cases but no asymptomatic ones are observed (red and blue shaded histograms, respectively, in *SI Appendix*, Fig. S12). Testing only symptomatic cases can result in at least a fourfold increase in the IFR that is calculated. Limitations in testing capacity may also impact the estimated IFR. If the testing capacity is limited at the start of the outbreak, the observed IFR measured during the epidemic (the orange vertical line in *SI Appendix*, Fig. S12) will be higher than the IFR expected if all symptomatic cases were tested. Variation in individual model parameters within the range supported by the case and serology data does not result in substantial variation in the IFR calculated for each testing scenario.

## Discussion

With a transmission model that incorporates daily changes in RT-PCR testing capacity and is fit to observed case data and serology, we estimate that the probability that an exposed individual develops symptoms is low. Since asymptomatic infections represent a large fraction of the infected population, they contribute substantially to community transmission in the aggregate together with presymptomatic cases, even when they individually transmit at a low per capita rate. They also contribute substantially to building herd immunity.

We use testing information to estimate the probability that a new case will become symptomatic without the biases present in cruise ship (17) and traveler studies (18), or the parameter confounding present in citywide models. Early cruise ship and evacuee studies found that most COVID-19 cases were symptomatic. However, given the small number of total infections (18, 28), evacuee studies may overestimate the fraction of symptomatic cases if infections in observed severe cases (29) last longer (30) than in asymptomatic ones. Cruise ship studies may likewise overestimate this parameter if asymptomatic cases, which were tested later than symptomatic cases (17), recover prior to testing. Citywide models, which avoid these biases, indicate that most COVID-19 cases are undetected (9). They confound, however, the fraction of symptomatic cases with the reporting or hospitalization rate, as they neglect daily testing changes, and cannot distinguish between asymptomatic and undetected cases. The alternative approach of fitting the models to death data is not necessarily exempt from biases in parameter estimates, due to changes in hospital capacity over time (31, 32), comorbidities in host populations (33, 34), and the long delay between the onset of infection and death (35). Furthermore, the underreporting of cases can also bias the assumed case fatality rate (32). Our approach resolves these issues by incorporating daily testing capacity as part of the model when estimating parameters from serology and case data. Models without explicit consideration of this capacity have difficulty estimating the proportion of cases that are symptomatic from these data (36), suggesting that including testing is crucial.

If asymptomatic individuals transmit at a high rate, then the overall reproductive number preintervention in New York City is larger than the two to three range often assumed in models (6–8) and media reports (11, 37–39) based on early estimates from Wuhan (4, 40, 41). Furthermore, we find no supported parameter combinations in which both the overall and symptomatic reproductive numbers fall within this range. Early Wuhan models may underestimate $R_{0}$ by ignoring presymptomatic transmission and making restrictive assumptions, including that COVID-19 has the same incubation period and serial interval as SARS-CoV (4, 40, 41), or that most cases are symptomatic (42). Early Wuhan case data may be insufficient to precisely estimate $R_{0}$ without making these assumptions (43–45). Thus, models and intervention strategies should consider that the overall $R_{0}$ may be higher than three in certain locations (5, 46).

If asymptomatic individuals are unlikely to transmit and do so with low probability, then the small fraction of cases that are symptomatic are transmitting at a high rate, in line with recently reported “superspreading” events (47, 48). Superspreading events are instances in which a single infected individual infects a large number of people. These events can be hard to measure on a population level in the absence of detailed transmission data. In classic superspreading dynamics, most primary cases do not result in many secondary cases, while a subset of primary cases result in a large number of secondary cases (8, 49, 50). This heterogeneity in the reproductive number is, indeed, what we observe when asymptomatic individuals transmit poorly. Our model is admittedly a coarse description of this heterogeneity, since it incorporates only two different classes of infections, symptomatic or asymptomatic. Future models can build upon this framework with additional classes for age, socioeconomic status, location, or susceptibility (51), using fine-scale case data. These models could elucidate how infections in hospitals or home care settings may be contributing to the high $R_{0}$ of symptomatic cases. However, our results also indicate that, even when the symptomatic reproductive number is large, presymptomatic and asymptomatic infections contribute together to at least 50% of the overall force of infection.

It follows that community-wide interventions that account for nonsymptomatic cases should be crucial for mitigating outbreaks. If asymptomatic cases transmit poorly, then concurrent additional interventions targeting superspreading symptomatic infections may help reduce community transmission.

Resolving the nonidentifiability of the relative strength of asymptomatic transmission ($b_{a}$) would require extensive community testing and contact tracing of asymptomatic cases. Community testing on its own can provide an estimate of the total proportion of cases that are asymptomatic, but it may not provide insight on whether those asymptomatic individuals can transmit and how well they can transmit. Symptomatic and asymptomatic individuals have similar viral loads (52), but a high viral load does not necessarily imply high transmissibility. One limitation of early contact tracing studies is that estimates of transmissibility may oversample symptomatic index cases and contacts, particularly during the early phase of an epidemic (15, 53). In certain studies, only symptomatic contacts are further investigated. Ideally, one would use frequent systematic community testing for studies identifying both symptomatic and asymptomatic potential index cases for further contact tracing and testing of all contacts regardless of symptoms. Furthermore, fixing the probability that an infection becomes symptomatic based on the results of serology-informed models such as ours could increase the precision with which contact tracing studies can estimate the strength of asymptomatic transmission. Colleges that are currently reopening may be ideal test locations for this kind of combined approach, which may also help detect superspreading events.

While it cannot capture all testing intricacies, our framework illustrates how transmission models can incorporate daily changes in testing capacity and identify parameters that were previously difficult to estimate, such as the probability that an infection will become symptomatic. While we do not explicitly denote differences between laboratories, hospitals, or diagnostic tests, we account for this variation by including additional measurement noise after simulating the RT-PCR testing process. We also consider how sampling individuals without COVID-19 may deplete the daily testing capacity. In particular, hospitalized individuals with non–COVID-19–related severe respiratory disease may have a higher priority for testing than nonsevere COVID-19 cases. Our model uses syndrome surveillance reports (54–57) of respiratory illness from New York City hospitals in previous years, along with weekly influenza cases, to estimate the number of non–COVID-19 severe respiratory cases that were tested. The statistical model assumes that, in every year, only a fraction of influenza cases are confirmed and that noninfluenza respiratory cases exhibit seasonality. We use flu and syndrome surveillance estimates from previous years to estimate the fraction of influenza cases that are not confirmed and the shape of the seasonality on non–influenza-related respiratory illness. During the 2020 epidemic, COVID-19 mitigation measures that reduced urban mobility may have also reduced transmission of other respiratory diseases such as influenza. The model captures some of this decrease, since the number of severe non-COVID respiratory cases is a function of the number of confirmed flu cases in the same season. The model thus captures the impact of decreased flu cases in 2020 due to changes in mobility patterns. This framework could be used in conjunction with other epidemiological models, and extended to other municipalities or countries with location-specific testing priorities, retesting procedures, or diagnostic tests.

In cities where mobility information is available, the statistical model may include overall population mobility as a covariate. In other cities that report the daily number of hospitalized individuals with COVID-19 symptoms who were tested each day, one could subtract from this number the total COVID-19 hospitalizations estimated by the epidemiological model, to obtain the number of non–COVID-19–positive hospitalized cases that were tested. Depending on the information available for each location, future iterations of this framework could explicitly incorporate different diagnostic tests and their respective sensitivities and specificities. It could also be used to examine how altering testing strategies such as switching from symptom-based testing to community testing may improve transmission parameter inference and efficacy of control efforts. This may be an important consideration for countries that have limited testing capacity but are still in the midst of the first pandemic wave, such as India.

Given the potential role of population density and socioeconomic status on contact rates and access to care, there may be considerable heterogeneity in infection rates and seropositivity in different neighborhoods of New York City. While overdispersion in measurement error can implicitly account for this variation in our implementation, future formulations could do so explicitly with a spatial model of transmission between neighborhoods and within specific settings such as hospitals and home care networks. This level of resolution would require, however, observed cases, testing capacity, and hospitalizations within each unit. Incorporating human movement estimates into the model could enable analysis of how the infectious period of the virus may impact the clustering of cases within particular neighborhoods.

Future studies can investigate the impact of including a testing submodel on parameter estimation and the level of detail required in such a submodel. For example, one could compare the results of parameter estimation from fitting a given epidemiological model with a queue-based testing model to those that assume a fixed reporting rate and a delay in the reporting of cases. We expect the former to exhibit more uncertainty when informed by surveillance data from the beginning of the pandemic, when little testing capacity is available, but to reduce this uncertainty as the time series is extended and this capacity changes. Models that assume a fixed reporting rate may underestimate the range in uncertainty of epidemiological parameters that are heavily informed by the early part of the time series, and may even underestimate the values of the parameters themselves. Models with a queue-based testing submodel may obtain more-precise estimates of parameters that impact the end of the outbreak, such as those related to the depletion of susceptible individuals, acquisition of immunity, or, in our model, the impact of social distancing and stay-at-home orders on overall transmission. Even if including some form of testing model that takes into account changes in capacity is key to obtaining more precise parameter estimates, simpler versions of our implementation may be sufficient. For example, the more generalizable components, such as the testing of hospitalized individuals, may be more important than taking into account their resampling as they leave the hospital. Simplifying the testing model based on model selection analyses can facilitate wider adaption of the testing framework to other cities, countries, or time periods.

Future versions of the model could also capture heterogeneity in the severity of infection and the acquisition of immunity. When fitting the model, we treat seropositivity as a reasonable correlate of herd immunity. The assay used to measure seroprevalence in New York City (24) elicits neutralizing antibodies (58) and can detect seroconversion in severe, mild, and asymptomatic cases (58). We assume that this herd immunity does not decay over the course of the study, given the short duration of time from March to June 2020 and the observation that antibody responses can persist for at least 5 mo following the start of the pandemic in New York City (59). The immune response to the SARS-CoV virus consists of several components including antibody (60) and T-cell mediated (61) responses, and heterogeneity in particular pathways of the immune response can influence the severity of infections (62, 63). The severity of infection may, in turn, impact the type, strength, and duration of the immunity acquired (64, 65). As future experimental studies determine how each immune response can mitigate infection, viral shedding, and transmission, relevant aspects of the dynamics of host immunity can be incorporated into the model and corroborated with data. Understanding how host heterogeneity in immune responses may impact the infection severity and herd immunity may be valuable when considering long-term vaccination policies.

While a model with explicit within-host dynamics would be challenging to fit using case data, relevant aspects could be incorporated in several ways. For example, the model could include additional classes of infection corresponding to levels of severity. Alternatively, a distribution of susceptibility or immunity could be used to capture heterogeneity in the immune response between individuals. Finally, the model could incorporate functional forms for the acquisition and waning of immunity that are fixed based on experimental observations of serology and T-cell dynamics. Fitting these models to times series from multiple locations will improve inference, but the testing capacity and strategy in each location should be taken into account when doing so.

Our finding that many individuals were already infected by March 1 is consistent with earlier estimates that community transmission began in February in New York City (27, 55, 66). Previous studies could not explain, however, why no substantial increase in COVID-19–like illness was observed prior to February 28 in syndrome surveillance data (55). Our simulations show that the lag between infection onset and hospitalization can explain this discrepancy. Even when initialized with many infected cases on March 1, simulated hospitalizations do not rise until several weeks later, concurrent with observed COVID-19 hospitalizations (Fig. 5). Most likely, the estimated initial conditions suggest multiple parallel foci of initiation of the epidemic with multiple importations of infections. Another suggested possibility is a dosage dependence effect, wherein the severity of an individual’s infection depends on the size of the virus population that the person becomes infected with during one or more transmission events, and hence on the overall viral load of COVID-19 in the community. In this scenario, early COVID-19 cases in February and early March would be less severe. This would be consistent with the syndrome surveillance data, where we see a rise, in early March, of respiratory infection reports in the emergency departments of hospitals, but do not yet see a rise in COVID-19 hospitalizations. This phenomenon might also explain why our model slightly underestimates the peak in daily hospitalizations, even though it correctly identifies the time and shape of that peak.

We show that testing capacity and strategy can substantially affect estimates of the IFR, a quantity that is frequently used by public health officials in assessing the severity of an outbreak. Our model ignores several factors, such as nonhospital deaths from COVID-19, which may increase the true IFR, and rising trends in hospital capacity and improved treatments, which may decrease it. Nevertheless, our results underscore the importance of considering testing strategy and capacity when interpreting literature estimates of the IFR.

In conclusion, explicit consideration of changes in testing capacity allows us to infer with certainty, from case and serology data, that most new COVID-19 cases do not become symptomatic. We also inferred that the overall or symptomatic reproductive number may be larger than often assumed, depending on how well asymptomatic cases can transmit. Despite this uncertainty, the strong consistent contribution to community transmission from cases without symptoms observed across scenarios supported by the data should be considered when formulating public health intervention strategies. Making available detailed information on testing policy and data on testing capacity over time will strengthen the ability of epidemiological models to learn from the past and inform us about the future.

## Materials and Methods

We examine three different model structures that have been used to characterize COVID-19 dynamics in previous studies (Fig. 1). All models are modified versions of the traditional SEIR model (67). The first model, the SEPIR model (17, 68), is the most standard extension in which transitions are between a linear chain of compartments. Its formulation adds a compartment P for presymptomatic transmission. The second one, the SEIAR model (7, 9), differs conceptually in that it includes asymptomatic individuals rather than presymptomatic ones, and defines them as distinct, in the sense that they will never transition to exhibiting symptoms. This definition implicitly recognizes that there are essentially two classes of individuals in terms of susceptibility to disease and symptoms. The third structure for the SEPIAR model (6, 26) is a combination of the first two and includes them as nested, particular, cases.

All three models include a chain of m exposed classes to incorporate the total time between the onset of infection and the onset of symptoms as gamma distributed (with mean 5.5 d and standard deviation 2.25 d) (69). Symptomatic individuals are subdivided into two sequential classes, $I_{S_{1}}$ and $I_{S_{2}}$, for practical purposes, to follow their numbers before and after some of them transition to hospitalization. Individuals spend an average of 1/ϕ_*S*_ days in $I_{S_{1}}$ and 1/γ days in $I_{S_{2}}$.

The parameter $R_{0}$ represents the reproductive number experienced by symptomatic individuals. We define a baseline preintervention transmission rate in symptomatic individuals, $\beta_{0}$, by dividing $R_{0}$ by the average total time that nonsevere cases transmit with symptoms. We also define a postintervention transmission rate $\beta_{1}$, which is equal to the preintervention transmission rate $\beta_{0}$ multiplied by a scaling factor $b_{q}$. Low values of $b_{q}$ represent a substantial reduction in the transmission rate due to interventions. Social distancing guidelines were issued by New York City starting on March 17 (70, 71), and a stay-at-home order was issued which took effect on the evening of March 22 (72). Thus, prior to the imposition of social distancing, the transmission rate of symptomatic individuals in our models, β(t), is equal to $\beta_{0}$. From March 18 through March 22, β(t) decreases linearly from $\beta_{0}$ to $\beta_{1}$. From March 23 onward, β(t) is equal to $\beta_{1}$.

In all models, a fraction $p_{S}$ of exposed individuals $E_{m}$ become symptomatic. After an average of 5 d of transmission, symptomatic cases are hospitalized with probability $p_{H}$. Symptomatic cases that are not severe enough to require hospitalization recover at rate γ. Hospitalized individuals recover at rate *h*_*v*_ = 1/13 (30) and do not transmit while hospitalized. In practice, some individuals with severe COVID-19 symptoms that required hospitalization may not have been hospitalized, due to barriers to care. However, their contribution to community transmission is unlikely to have been substantial. These individuals would remain isolated at home during the period of severe infection and avoid nonhousehold contacts, while household contacts would have been exposed for a substantial time prior to the onset of severity. We assume a fixed population size for New York City of 8 million individuals (73).

Assumptions about which infected classes are infectious and how they contribute to the transmission rate allow us to reduce the SEPIAR model to the SEPIR or SEIAR models. Presymptomatic individuals transmit for an average of about a day [0.92 d (25)] at a transmission rate equal to the baseline transmission rate β(t) multiplied by a scaling factor *b* = $b_{p}$. Asymptomatic infections transmit for an average of 5 d, equal to the average duration between the onset of symptoms and hospitalization in severe cases, at a transmission rate equal to the baseline rate β(t) multiplied by scaling factor $b_{a}$.

The models are implemented numerically via an Euler approximation of the deterministic equations to which demographic stochasticity is added. Specifically, the number of individuals making state transitions from compartments with more than one exit is drawn from an Euler-multinomial distribution (74). The number of individuals making state transitions from compartments with only one exit is drawn from a binomial distribution.

### Description of Testing Model.

The model takes into account daily changes in the testing capacity, using estimates of daily tests conducted in New York City from the New York State Department of Health (23), as well as the retesting of severe and nonsevere symptomatic cases prior to leaving the hospital or quarantine. We assume that there are two categories of cases—severe (hospitalized) cases and nonsevere cases subject to different testing priorities (75): the initial testing of new hospitalized COVID-19 cases (highest priority), the retesting of those individuals when they leave the hospital, the testing of new nonsevere symptomatic COVID-19 cases, and, finally, the retesting of those symptomatic cases (lowest priority). All severe COVID-19 cases after March 1 are sampled when they enter the hospital and eventually tested once enough capacity is available. We assume that symptomatic nonsevere cases are sampled at the same time, in the course of their infection, as severe cases. However, we assume that they are not tested if they recover before enough testing capacity is available. During the early stages of the epidemic, the CDC recommended test-based strategies to determine when to conclude home isolation or hospitalization (76). Accordingly, we assume that hospitalized cases are retested twice (over a 24-h period) after the average length of time in the hospital (13 d), while nonsevere cases are likewise retested twice after the end of a 14-d quarantine period.

We also take into account the potential for non–COVID-19 severe respiratory cases to be sampled in hospitals and tested (with the same priority as hospitalized COVID-19 cases). We use confirmed influenza cases (77) and syndrome surveillance reports of respiratory disease from emergency departments in New York City hospitals in previous years (78) to estimate the number of non–COVID-19 severe respiratory cases that may have been sampled (*SI Appendix*). We assume that the RT-PCR testing has a sensitivity of 90% (79), that testing takes 48 h (80), and that there is an additional negative-binomial distributed dispersion after the RT-PCR testing with standard deviation $\sigma_{M}$. This dispersion takes into account variation in sampling and testing protocols across laboratories and hospitals, as well as variation in the sensitivity and time required for different PCR assays.

### Overview of the Model Fitting and Inference Strategy.

Unless otherwise mentioned, we fit the following parameters: the recovery rate for nonsevere symptomatic infections (γ); the scaling factors for asymptomatic, presymptomatic, and postintervention transmission ($b_{a}$, $b_{p}$, and $b_{q}$); the symptomatic probability ($p_{S}$) and the hospitalization probability ($p_{H}$); the reproductive number for symptomatic cases ($R_{0}$); the dispersion parameter for RT-PCR testing ($\sigma_{M}$); and the initial number of infected ($I_{0}$) and exposed ($E_{0}$) individuals at the start of the simulation on March 1, 2020. We use the iterated filtering algorithm MIF (81) within the R-package POMP (for partially observed Markov process models) to fit parameter combinations by likelihood maximization. The iterated filtering algorithm is specifically designed for fitting stochastic and nonlinear models with hidden variables in the presence of both process and measurement error. We apply the sequential Monte Carlo algorithm pfilter (82) to evaluate the likelihood of the final parameter combinations obtained with the computational search. Likelihoods are estimated by simulating state variables at each observation time from an underlying Markov process model, and then calculating the likelihood of each observation given the simulated value of the state variable and a measurement model. For the analysis of the full SEPIAR model, we generate a Monte Carlo profile (83) for the relative strength of asymptomatic transmission ($b_{a}$).

For all resulting parameter combinations within two log-likelihood units of the maximum-likelihood estimates (MLE), we then calculate the likelihood with respect to serology, using seroprevalence data previously published by ref. 24 from a screening group representative of the general population, using plasma samples from patients at Mount Sinai Hospital in New York City. In the Mount Sinai study, random, deidentified, and cross-sectional samples were obtained over the course of the outbreak from patients at obstetrics and gynecology visits and deliveries and oncology-related visits, as well as hospitalizations due elected or planned surgeries, transplant surgeries, preoperative medical assessments, and related outpatient visits, cardiology office visits, or other regular office or treatment visits whose purpose was unrelated to COVID-19 (24). The assay used to measure seroprevalence (24) elicits neutralizing antibodies (58) and can detect seroconversion in severe, mild, and asymptomatic cases (58). We treat the seroprevalence measurement at each time point as a measure of short-term herd immunity in the population, specifically, of the proportion of the population that has already recovered from COVID-19 infection. We assume that this herd immunity does not decay over the course of the study, since antibodies have been shown to persist for at least 5 mo (59). We compare the seroprevalence at each time point from the serology data to the recovered fraction of the population *R*/*N* from simulated trajectories of the epidemiological model. When calculating the likelihood of each trajectory at each observation time with respect to the seroprevalence data, we assume that the number of people who seroconverted in the Mount Sinai study is drawn from a binomial distribution with p equal to the value of *R*/*N* in the simulated trajectory at that time, and N equal to the total number of people sampled. We sum the log-likelihoods across all observation time points and then average over all trajectories using the logmeanexp function in the R package POMP (82) to obtain a log-likelihood for each parameter combination with respect to the serology data. We isolate all combinations supported by the serology data that have log-likelihoods within two units of the MLE.

For each combination, we examine the proportion of cases that are symptomatic, $p_{S}$, the reproductive number in symptomatic individuals, $R_{0}$, and the overall reproductive number for the model, $R_{0_{NGM}}$. We derive the following expression for $R_{0_{NGM}}$ using the next-generation matrix (84):

$$R_{0_{NGM}}=\frac{\beta *b_{p}}{\phi_{U}}+\frac{\beta *b_{a}\left(1-p_{S}\right)}{\phi_{S}}+\frac{\beta p_{S}}{\phi_{S}}+\frac{\beta \left(1-p_{H}\right)p_{S}}{\gamma }.$$ [1]

### Calculation of the IFR.

The IFR is frequently defined as the ratio of deaths to cases. Let $IFR_{all}$ represent the IFR with respect to all cases,

$${\text{IFR}}_{\text{all}}=\frac{\text{Confirmed deaths}}{\text{All cases}}.$$ [2]

This is equivalent to the proportion of all cases that result in death. We can estimate this quantity using the parameters from the fitted SEPIAR model. Recall that $p_{S}$ is the probability that a case is symptomatic, and $p_{H}$ is the probability that a symptomatic case becomes hospitalized. These quantities are equivalent to the proportion of all cases that are symptomatic, and the proportion of symptomatic cases that are hospitalized. Let $p_{F}$ represent the proportion of all hospitalizations that are fatal. Since this parameter is not fitted in the SEPIAR model, we estimate it from observed data by dividing the total number of confirmed COVID-19 deaths by the total number of confirmed COVID-19 hospitalizations in New York City during the study period. We use data updated on June 15, 2020 from the New York City Health Department COVID-19 Data Portal (22), and obtain an estimate of $p_{F}=0.33$. We can then write an expression for ${\text{IFR}}_{\text{all}}$ using the parameters estimated with the fitted SEPIAR model,

$${\text{IFR}}_{\text{all}}=p_{S}p_{H}p_{F},$$ [3]

and obtain this quantity for each parameter combination supported by case and serology data (red histogram in *SI Appendix*, Fig. S12).

Let ${\text{IFR}}_{symp}$ represent the IFR that would be estimated if all symptomatic cases were observed but no asymptomatic ones were observed. This is equivalent to the proportion of all symptomatic cases that result in death,

$${\text{IFR}}_{\text{symp}}=\frac{\text{Confirmed deaths}}{\text{All symptomatic cases}}=p_{H}p_{F}$$ [4]

(shown in the blue histogram of *SI Appendix*, Fig. S12).

We compare these two quantities with the observed IFR, calculated by dividing the total deaths by the total number of PCR-confirmed COVID cases in New York City during the study period (the orange line in *SI Appendix*, Fig. S12).

### Additional Details.

Further details of the SEPIAR equations, testing model, Monte Carlo profile of the SEPIAR model, initial grid searches, and model comparison of the SEPIR and SEIAR models, and derivation of the overall reproductive number $R_{0_{NGM}}$, are provided in *SI Appendix*.

## Supplementary Material

Supplementary File

## Data Availability

Code used to simulate and fit the epidemiological model has been deposited in Github at https://github.com/pascualgroup/COVID_NYC_Epi_Model (85).
